# The representation of speech conversations in the human auditory cortex: role of social and semantic factors

**DOI:** 10.1093/cercor/bhag023

**Published:** 2026-03-11

**Authors:** Etienne Abassi, Robert J Zatorre

**Affiliations:** The Neuro (Montreal Neurological Institute-Hospital), Mcgill University, 3801 Rue University, Montreal, QC H3A 2B4, Canada; Centre for Research in Brain, Language and Music (CRBLM), 2001 Av. McGill College, Montreal QC H3A 1G1, Canada; International Laboratory for Brain, Music and Sound Research (BRAMS), Université de Montréal, 90 Av. Vincent D'Indy, Outremont, QC H2V 2S9, Canada; The Neuro (Montreal Neurological Institute-Hospital), Mcgill University, 3801 Rue University, Montreal, QC H3A 2B4, Canada; Centre for Research in Brain, Language and Music (CRBLM), 2001 Av. McGill College, Montreal QC H3A 1G1, Canada; International Laboratory for Brain, Music and Sound Research (BRAMS), Université de Montréal, 90 Av. Vincent D'Indy, Outremont, QC H2V 2S9, Canada

**Keywords:** fMRI, predictive coding, semantic context, social context, speech perception

## Abstract

Human social nature has shaped auditory perception, as hearing is essential for navigating social interactions, especially when listening to others’ conversations. While much research has examined how the brain processes isolated words or sentences, far less is known about how broader social and semantic contexts influence speech comprehension. We used 7 T fMRI to examine neural responses while participants listened to two-speaker dialogues versus single-speaker monologues, presented either in intact or sentence-scrambled order. Twenty-four healthy young adults listened to AI-generated five-sentence conversations designed to independently manipulate social (dialogue vs. monologue) and semantic (intact vs. sentence-scrambled) contexts. Whole-brain univariate analyses revealed increased activity for scrambled compared to intact conversations in the left superior temporal sulcus (STS), consistent with predictive-coding models. Although social context alone showed no main effect, an interaction emerged: semantic disruption elicited stronger responses in dialogues than monologues within the STS. Multivariate pattern analyses further revealed higher classification accuracy of individual sentences within dialogues vs. monologues, particularly in the left anterior STS and inferior frontal gyrus, suggesting that social context enhances linguistic encoding. Together, these findings indicate that the left STS integrates both semantic and social information, supporting predictive and context-sensitive mechanisms crucial for real-world verbal communication.

## Introduction

Human conversations are at the core of our social world. Much of our interaction involves not only speaking but also listening to others, helping us gather information and understand our social environment. While extensive research explored speech perception at the level of isolated words or sentences (Hickok and Poeppel 2007; Golestani et al. 2009, 2013; Zekveld et al. 2011, 2012, 2013; Roland et al. 2012; O’Neill et al. 2021), the neural mechanisms underlying processing at the scale of full conversations remain underexplored. Unlike isolated sentences, conversations unfold over time, involve multiple speakers, and are shaped by both semantic content and social dynamics (Pickering and Garrod 2021). Understanding how these elements are integrated in the brain is essential for a comprehensive account of naturalistic speech processing.

Recent neuroimaging work in the visual domain has shown that social interaction modulates perceptual encoding even when low-level features are matched. More specifically, Abassi and Papeo (2020) demonstrated that representations of individual bodies are sharpened when the two are depicted as interacting face-to-face vs. back-to-back. By analogy, we tested whether conversational context, defined by alternating speakers engaged in turn-taking, sharpens neural encoding of speech. To do so, we used the same stimuli and protocol as in our recent behavioral study (Abassi and Zatorre 2025), where we showed that both semantic and social context improved comprehension of speech in noise; the present study investigates the neural mechanisms underlying that effect.

Semantic context plays a critical role in facilitating speech comprehension. Studies show that listeners use semantic coherence to predict and understand incoming speech, particularly in real-world settings where input is continuous and noisy (Cox et al. 1987; Baker and Hazan 2010, 2011; Broderick et al. 2019). This facilitation has been framed within predictive coding models, where higher-level semantic representations generate expectations that constrain the interpretation of sensory input (Friston 2005; Clark 2013). In particular, the superior temporal sulcus (STS) has been implicated in integrating such semantic information with acoustic signals (Lau et al. 2008; Sohoglu et al. 2012). As a multimodal hub (Michon et al. 2022; Kausel et al. 2024), the STS receives input from auditory, visual, and language-related areas and supports the interpretation of socially relevant cues like gaze, prosody, and speaker identity (Allison et al. 2000; Redcay 2008). However, how the STS supports comprehension across extended conversational scales is less well understood.

Social context adds another dimension to speech comprehension. Here, by social context we refer to the interactional structure of communication and, specifically, the presence of multiple speakers engaged in turn-taking and reciprocal exchange. Dialogues involve dynamic interactions, such as turn-taking, feedback, and adaptation, that are absent in monologues (Pickering and Garrod 2004, 2021; Branigan et al. 2011; Menenti et al. 2012). Despite the important role of interactions across speakers, most neuroimaging studies on speech focus on single-speaker speech (eg Lerner et al. 2011; Brennan et al. 2012; Brennan and Hale 2019), largely neglecting the neural impact of social dynamics. However, humans are highly attuned to social cues, and temporal-voice areas within the STS respond selectively to vocal signals and social information (Belin et al. 2000; Levy et al. 2003). Thus, dialogues likely engage additional cognitive and neural resources, requiring listeners to track both content and interpersonal dynamics. In the visual domain, the presence of interacting agents has been shown to enhance perceptual and neural processing of these agents in body-selective regions within the lateral occipital cortex (Abassi et al. 2020; Bellot et al. 2021). In the auditory domain, we recently found that dialogues, compared to monologues, facilitated speech-in-noise processing, while intact sentence order improved perception relative to scrambled order (Abassi and Zatorre 2025). These findings suggest that listeners leverage both social and semantic cues, yet how these factors jointly influence brain activity remains an open question.

To address this gap, we used 7 T fMRI to examine how social and semantic contexts influence speech processing. Participants listened to AI-generated sentences varying along two dimensions: social context (dialogue vs. monologue) and semantic context (intact vs. sentence-scrambled). We conducted univariate analyses to test whether activity in language-selective regions was modulated by these factors, and multivariate analyses to test whether sentence representations were sharpened in context. Building on evidence that the left STS integrates both semantic and socially relevant cues, we hypothesized that this region would be particularly sensitive to our manipulations. By combining methodological approaches, we aim to clarify how the brain supports the interplay between semantics and social interaction that defines real-world conversation.

## Materials and methods

### Participants

24 healthy volunteers (12 females, 12 males; mean age 26.3 years; SD = 4.1) participated in our study. All were native English speakers with normal hearing and reported no history of psychiatric or neurological disorders. All were screened for MRI contraindications and provided written informed consent. The study was approved by the McGill University Faculty of Medicine Institutional Review Board (protocol A08-B75-22B/22–07-116, approved 2022 October 3).

### Conversational stimuli

The stimuli were a subset of those used in our previous behavioral study (Abassi and Zatorre 2025), available online (https://osf.io/vqs7h/). Sample audio for each condition is provided as supplementary material (Audio 1–4).

**Figure 1:**
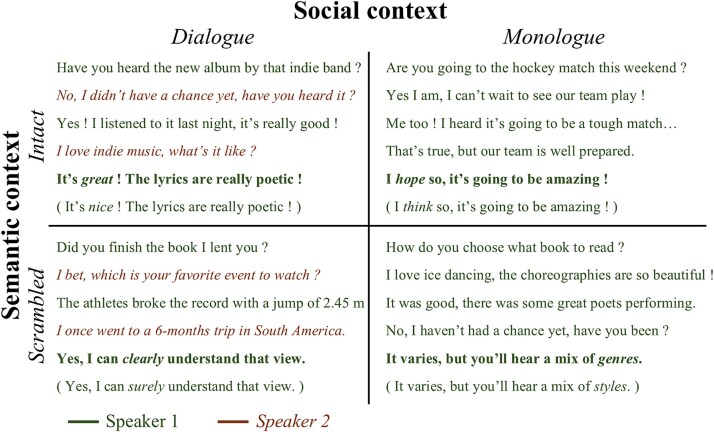
Example of conversations with a 2x2 design with factors social context (dialogue/monologue) and semantic context (intact/sentence-scrambled). The fifth sentences in bold represent the target sentences for MVPA decoding. The sixth sentences in parenthesis represent the probes used as attentional catch, that could be exactly the same or one-word different from the fifth sentences.

The set (Fig. 1) comprised English two-speaker dialogues and single-speaker monologues (social context) arranged in intact or sentence-scrambled order (semantic context). All texts were generated with ChatGPT (GPT-3.5, OpenAI) and converted to speech using Google Text-to-Speech. Dialogues featured alternating speakers, providing interactive dynamics absent in monologues, allowing us to isolate effects of social interaction. Extended details on stimulus creation appear in Abassi and Zatorre (2025).

Stimuli consisted of two sets of eight 5-sentence conversations (16 unique sequences; eight were used for half of the participants and the other eight for the other half of the participants), selected from a larger corpus of 35 conversations based on five themes (Music, Cinema, Sport, Literature and Travel). These specific 16 conversations were selected because they produced the strongest behavioral differences between dialogues and monologues in our prior study. Each conversation was paired with two probe sentences that were either identical to or differed by one word from the fifth (target) sentence. Probes served as catch trials to ensure attention. The altered word position varied randomly to avoid predictability. The substituted words were chosen to be semantically congruent but to differ phonologically from the original, while avoiding semantic or pragmatic violations.

The sentences within conversations were arranged as follows. For dialogues, half began with the female and half with the male voice; monologues used one consistent voice per sequence. Scrambled versions were created by randomizing sentence order across and within conversations, excluding the fifth sentence, ensuring no residual conversational structure (eg question–answer pairs).

Crucially, the linguistic content was identical across social conditions. The very same five-sentence sequences appeared in both the dialogue and monologue versions; the only difference was whether the sentences were produced by alternating speakers (dialogue) or a single speaker (monologue). Speaker identity for the fifth (target) sentence was counterbalanced across items and participants to prevent any systematic bias tied to a particular voice. Thus, any social-context effects cannot arise from differences in lexical content, sentence semantics, or syntactic complexity, which were perfectly matched across conditions by design.

In total, 64 conversational stimuli were used (16 per condition), each separated by 350 to 550 ms gaps (800 ms before probes). The full conversation plus probe averaged 18 s in duration.

### Single-sentence stimuli

In addition to the conversational stimuli, we created a complementary set of single-sentence stimuli corresponding to the fifth sentence from each 5-sentence conversation. These were presented in isolation, without conversational context. Each was followed by a probe sentence that was either identical or differed by one semantically congruent word. A silent 800-ms gap separated the sentence and probe, yielding an average total duration of 6 s. These isolated sentences served as training data for the multi-voxel patterns analyses (MVPA) classifier.

### Design of the main fMRI experiment

The experiment comprised two parts: the main fMRI task, and a speech functional localizer task (described below). In the main task, dialogues and monologues, in both intact and sentence-scrambled orders, were presented in random order across six runs. Half of the conversations were repeated 3 times over 3 runs (one presentation by run), while the other half was repeated 3 times over of 3 others runs. Each run included 16 blocks (20 s. each), comprising one five-sentence conversation plus its probe (~18 s.), and a 2 s. response window. Participants indicated whether the probe sentence was identical or different from the target sentence via button press with the right hand. This task was used to maintain attention and engagement. Each run began with a gradient-stabilization block (5.16 s) and ended with a cool-down block (12.04 s). Within runs, inter-block intervals were jittered (3.44–6.88 s; total 77.4 s) to decorrelate the hemodynamic response (Dale 1999). Total duration for each conversation run was 6.9 min.

Two additional runs contained only the single-sentence stimuli (the fifth sentences from each conversation). Each of these runs included 24 blocks (8 s. each) in which participants performed the same identity judgment task. Each sentence was repeated three times per run (six repetitions total). Timing and jitter parameters matched those of the main task (gradient stabilization = 5.16 s; cool-down = 12.04 s; jitter = 3.44 to 6.88 s; total 116.96 s inter-block interval). Each single-sentence run lasted 5.4 min.

Conversation and single-sentence runs were pseudo-randomly ordered, avoiding two consecutive single-sentence runs. Throughout the experiment, participants fixated on a white cross against a gray background while listening to the auditory stimuli.

### Speech functional localizer task

Prior to the eight experimental runs, participants completed an auditory speech functional localizer task for which presentation script and stimuli were taken from Scott et al. (2017) and used in recent studies (eg Olson et al. 2023; Wolna et al. 2024). Participants listened to 18-s blocks of intact or degraded speech. The Intact condition comprised spoken-English audio clips (eg interview segments), whereas the Degraded condition used acoustically matched but unintelligible, noise-vocoded versions (see Scott et al. 2017 for details). During the task, participants passively fixated a black dot on a white background. Four speech blocks were followed by a 14-s silent fixation block, and additional fixation blocks occurred at the beginning and end of each run (five per run total). Each participant completed one localizer run (~6.1 min) containing 16 speech blocks (eight intact, eight degraded). The localizer was used to identify brain regions responsive to speech perception, providing independent (regions of interest) ROIs for subsequent analyses of semantic and social-context effects (see below).

### Data acquisition

MRI data was acquired with the Siemens Magnetom 7 Tesla scanner installed at the McConnell Brain Imaging Centre of the Montreal Neurological Institute and Hospital, using a 32-channel head coil. Functional MRI data during task performance were acquired using multi-echo planar imaging (EPI) sequences with the following parameters: TR = 1.72 s, TE1 = 11.2 ms, TE2 = 27.8 ms, TE3 = 44.4 ms, 75 slices, slice thickness = 1.9 mm, no gap, field-of-view = 201.5 mm, flip angle = 67°, matrix size = 118 × 118, GRAPPA (phase encode and slice direction) acceleration factor 3, PE = AP. Structural images were recorded using an MP2RAGE T1 protocol with 0.7 mm isotropic resolution, TR = 5 s, TE = 2.92 ms, TI1 = 0.9 s, TI2 = 2.275 s, field-of-view = 226 × 220 mm, flip angle 1 = 4°, flip angle 2 = 4°, GRAPPA acceleration factor 3. The acquisition of two field maps was performed at the beginning of the fMRI session.

### Preprocessing

All preprocessing steps were carried out using fMRIPrep 23.2.1 using default parameters (Esteban et al. 2019), which is based on Nipype 1.8.6 (Gorgolewski et al. 2011). Briefly, each participant’s T1-weighted image was corrected for intensity non-uniformity (Tustison et al. 2010) and skull-stripped with antsBrainExtraction (ANTs 2.5.0; (Avants et al. 2008)). Brain tissue (CSF, WM, GM) was segmented using fast (Zhang et al. 2001) and spatially normalized to MNI152NLin2009cAsym (Fonov et al. 2009) via nonlinear registration (ANTs). For each BOLD run, head motion was estimated and corrected using mcflirt (FSL, (Jenkinson et al. 2002)), and the reference volume was co-registered to the T1w image with a boundary-based registration cost function (Greve and Fischl 2009). Confound regressors, including framewise displacement (Power et al. 2014), DVARS, global signals, and CompCor components (Behzadi et al. 2007), were extracted to model noise sources. Additional nuisance signals were derived from edge voxels (Patriat et al. 2017). Data were resampled in one interpolation step by composing all transformations, with nitransforms and cubic B-spline interpolation. Full details of the methods and parameters are provided in the fMRIPrep documentation. Multi-echo images were then processed and combined with tedana (DuPre et al. 2021) for T2*-based denoising, using default parameters. An adaptive mask reflecting echo quality guided T2 and S0 estimation^*^ (log-linear regression) and optimal combination (Posse et al. 1999). The resulting data underwent PCA (Li et al. 2007) and ICA, with components classified as BOLD (TE-dependent) or non-BOLD (TE-independent) via Kundu’s decision tree (Kundu et al. 2013). Standard Python libraries (numpy, scipy, pandas, scikit-learn) were used for computation, and the Dice similarity index (Dice 1945) evaluated spatial overlaps. Final steps included spatial smoothing with a Gaussian kernel of 6 mm FWHM for univariate analysis, and of 2 mm FWHM for multivariate pattern analyses (MVPA) using SPM 12 (Friston et al. 2007).

### Whole-brain univariate analyses

Whole-brain analyses were conducted in SPM 12 (Friston et al. 2007). The BOLD signal for each voxel in each participant was modeled in a random-effects general linear model including four regressors for the experimental conditions (intact dialogues, sentence-scrambled dialogues, intact monologues and sentence-scrambled monologues), a baseline regressor (inter-block intervals), and six motion parameters as nuisance covariates. A whole-brain repeated-measure ANOVA was performed with two within-subjects factors: social context (Dialogue/Monologue) x semantic context (Intact/Scrambled). Planned contrasts tested: Dialogues > Monologues (main effect of social context); Scrambled > Intact (main effect of semantic context); [(Scrambled dialogues > Intact dialogues) > (Scrambled monologues > Intact monologues)] (interaction between the two factors). Group-level effects were thresholded at *P* < 0.05, FWE corrected at the cluster level (voxelwise *P* < 0.001).

### Definition of regions of interest and ROI univariate analysis

**Figure 2:**
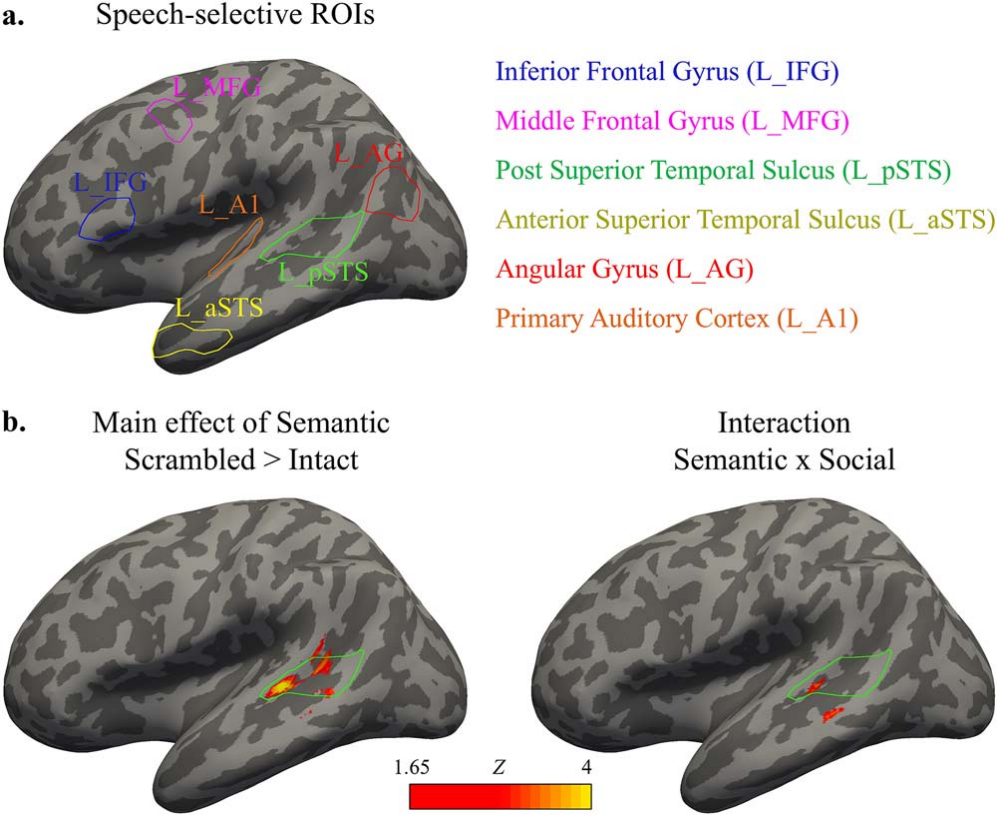
a) Functionally localized speech-selective regions. b) Whole-brain main effect of semantic context and interaction between semantic and social context.

We tested whether the context-related modulations observed at the whole-brain level occurred within speech-selective regions of the frontotemporal language network, as reported in prior work (Scott et al. 2017; Lee et al. 2024). These regions (Fig. 2a) were individually localized in each participant using the independent speech functional localizer described above, exactly following the procedure of Scott et al. (2017). Group-level masks were obtained from that study for six left-hemisphere regions: inferior frontal gyrus (L_IFG), middle frontal gyrus (L_MFG), posterior superior temporal sulcus (L_pSTS), anterior superior temporal sulcus (L_aSTS), and angular gyrus (L_AG). For each participant, localizer data was modeled with a GLM including regressors for intact and degraded speech, baseline, and motion parameters. Within each group-level mask, we identified voxels showing Intact > Degraded speech activity (*P* < 0.05) and selected the top 10% as that participant’s functional ROI. To assess specificity, we also included the left primary auditory cortex (L_A1; TE 1.0, Morosan et al. 2001) using the JuBrain Anatomy Toolbox (Eickhoff et al. 2005). Here, the top 10% voxels showing (Intact + Degraded) > Baseline were selected. Right-hemisphere homologues (R_IFG, R_MFG, R_pSTS, R_aSTS, R_AG, R_A1) were defined by mirroring the left-hemisphere masks and applying the same voxel selection procedure. From each ROI, mean β-values (relative to baseline) for each condition were extracted and entered into a 2 × 2 repeated-measures ANOVA (Social Context × Semantic Context) using Statistica (StatSoft).

### Classification of individual sentences within conversations with multi-voxel patterns analyses

MVPA assessed whether the context manipulations influencing behavioral performance in our previous auditory study (Abassi and Zatorre 2025) also modulated neural representations of speech. Following prior work in the visual domain (Abassi and Papeo, 2020), we reasoned that if dialogue structure sharpens perceptual encoding, then distributed neural patterns for individual sentences should be more discriminable in dialogues than in monologues. Thus, the MVPA tested whether our previously found behavioral facilitation was mirrored in neural representational space. Analyses were conducted within the ROIs defined above. For each participant and ROI, we estimated 24 multivariate β-patterns for the target (fifth) sentences of each condition (96 total patterns) and 48 β-patterns for the same sentences presented in isolation during the single-sentence runs. All β-patterns were run-wise normalized to prevent within-run correlations (Lee et al. 2018). A support vector machine (SVM; LIBSVM, Chang and Lin, 2011) implemented in CosmoMVPA (Oosterhof et al. 2016) was trained on eight sentence classes from the isolated single-sentence runs (six samples per class) and tested on the 24 patterns corresponding to the intact dialogue condition, using a one-against-one voting strategy. Each test iteration assigned one target-sentence pattern to one of the eight trained classes, and classification accuracy was averaged across iterations (chance level = 12.5%). This training–testing procedure was then repeated for the all the other three conditions. Classification accuracies were finally compared against chance (one-tailed t-tests) and then submitted to a 2 × 2 repeated-measures ANOVA (Social Context × Semantic Context) for each hemisphere and ROI.

## Results

### Catch trial performance

Participants achieved an overall accuracy of 92.7% (±9.3 SD) on the catch trial task, with 94.6% (±8.7 SD) accuracy in the single-sentence runs and 92.0% (±10.2 SD) in the five-sentence conversation runs. This high level of accuracy was expected given the simplicity of the task for native English speakers. Overall, this strong performance indicates that participants were paying attention while listening to the stimuli.

### Whole-brain univariate effects of context

We first identified the brain regions modulated by our manipulation of social and semantic contexts using a whole-brain approach (Fig. 2b). A main effect of semantic context (ie higher activity for sentence-scrambled vs. intact conversations) was found in a cluster overlapping with the speech-selective left pSTS ROI (MNI peak coordinates = [−58–31 6]; peak *z* = 4.42; cluster size = 267; cluster *pFWE* = 0.027), and in a cluster peaking within the right superior parietal lobe (MNI peak coordinates = [35–62 53]; peak *z* = 4.19; cluster size = 309; cluster *pFWE* = 0.017). We didn’t find clusters showing main effects of social context (ie higher activity for dialogues vs. monologues). However, interestingly we found a trend for an interaction between these two factors in a cluster overlapping with the speech-selective pSTS (MNI peak coordinates = [−58–35 2]; peak *z* = 3.52; cluster size = 84; cluster *pUNC* = 0.075). Univariate second level analyses maps and preprocessed fMRI data are available online at https://osf.io/vqs7h/.

### Univariate effects of context in speech-selective ROIs

**Figure 3:**
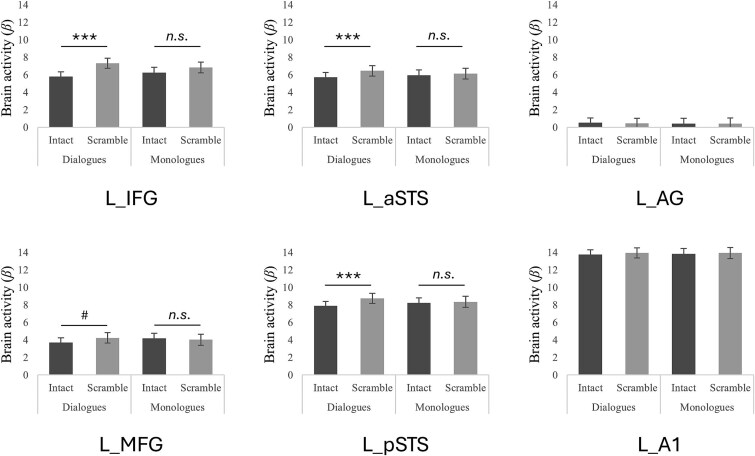
Univariate ROIs analyses within the functionally localized L_IFG, L_MFG, L_aSTS, L_pSTS, L_AG, L_A1. ^***^P < 0.001; # *P* < 0.01; *n.s. P* > 0.01.

Whole-brain effects of context were found in regions overlapping with speech-selective regions in the STS. To further investigate this finding and overcome the limits of group analysis in the definition of anatomical-functional correspondences (Saxe et al. 2006), we examined the effect of contexts directly within regions of the functionally defined frontotemporal language system (Fig. 3; Table 1). We found no effects of social contexts in any of the ROIs. However, we found main effects of semantic contexts (higher activity for sentence-scrambled than intact conversations) in L_IFG, L_pSTS and L_aSTS. Most importantly, we also found interactions between these two factors (all of them demonstrating a higher difference of activity between intact and scrambled for dialogues than monologues) in L_IFG, L_MFG, L_pSTS and L_aSTS. We didn’t find any effects in L_AG or L_A1.

**Table 1:** Results of the 2 social (dialogues/monologues) X 2 semantic (intact/scrambled) ANOVAs within functionally localized left ROIs.

ROIs	Effects of social			Effects of semantic			Interactions		
	*F*(1,23)	*P*	*np2*	*F*(1,23)	*P*	*np2*	*F*(1,23)	*P*	*np2*
L_IFG	< 0.01	> 0.250	< 0.01	14.2	0.001	0.56	5.16	0.033	0.19
L_MFG	0.27	> 0.250	0.04	0.75	> 0.250	0.08	6.96	0.015	0.23
L_aSTS	0.06	> 0.250	0.01	11.51	0.003	0.38	5.65	0.026	0.2
L_pSTS	0.02	> 0.250	< 0.01	14.88	0.001	0.4	8.09	0.009	0.26
L_AG	0.1	> 0.250	0.01	0.06	> 0.250	< 0.01	0.1	> 0.250	0.01
L_A1	0.01	> 0.250	< 0.01	0.86	> 0.250	0.04	0.07	> 0.250	< 0.01

### Multivariate classifier results: Single-sentence representations as a function of the context

We first found that classifications of individual sentences within whole conversations were significantly higher than chance for many of the conditions in most of our localized regions, with the notable exception of the L_AG (for detailed results, see Table 2). These findings demonstrate that our multivariate decoding approach reliably captured sentence-level information across language-selective regions.

**Table 2:** Results of the *t*-tests for comparing MVPA decoding accuracies against (12.5%) chance within functionally localized ROIS.

ROIs	Dialogues				Monologues			
	Intact		Scrambled		Intact		Scrambled	
	*t*(23)	*P*	*t*(23)	*P*	*t*(23)	*P*	*t*(23)	*P*
L_IFG	2.43	0.023	3.4	0.002	1.79	0.087	1.03	0.315
L_MFG	2.89	0.008	2.34	0.028	1.67	0.108	2.58	0.017
L_aSTS	5.43	< 0.001	5.55	< 0.001	3.13	0.005	4.12	< 0.001
L_pSTS	4.65	< 0.001	5.3	< 0.001	5.02	< 0.001	5.06	< 0.001
L_AG	1.9	0.07	1.25	0.224	1.8	0.085	1.06	> 0.250
L_A1	4.03	0.001	4.1	< 0.001	2.34	0.028	3.84	0.001

**Figure 4:**
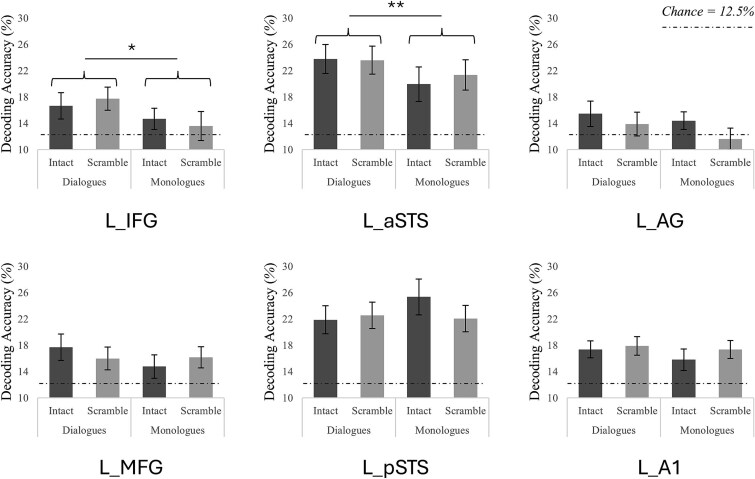
MVPA classification accuracy for cross-decoding of single sentences within conversations in the functionally localized ROIs (training on the multivariate patterns of the fifth sentences presented alone and testing when presented within whole conversations). ^*^P < 0.05; ^**^P < 0.01.

Critically, we also found main effects of social context (better classification for individual sentences within dialogues than within monologues) in L_IFG and L_aSTS but no effect of semantic context and no interaction in these regions (Fig. 4; Table 3). We didn’t find any other effects or interactions in the other ROIs (Fig. 4; Table 3).

**Table 3:** Results of the 2 social (dialogues/monologues) X 2 semantic (intact/scrambled) ANOVAs for the MVPA decoding accuracies within functionally localized left ROIs.

ROIs	Effects of social			Effects of semantic			Interactions		
	*F*(1,23)	*P*	*np2*	*F*(1,23)	*P*	*np2*	*F*(1,23)	*P*	*np2*
L_IFG	6.76	0.016	0.14	< 0.01	> 0.250	< 0.01	0.48	> 0.250	0.02
L_MFG	0.98	> 0.250	0.03	0.01	> 0.250	< 0.01	0.78	> 0.250	0.03
L_aSTS	8.04	0.009	0.08	0.08	> 0.250	< 0.01	0.14	> 0.250	0.01
L_pSTS	0.9	> 0.250	0.04	0.82	> 0.250	0.03	1.7	0.206	0.07
L_AG	1.35	> 0.250	0.06	1.17	> 0.250	0.09	0.19	> 0.250	0.01
L_A1	0.61	> 0.250	0.04	0.45	> 0.250	0.04	0.26	> 0.250	0.01

### Results in the right hemisphere

While the focus of our study was on the left hemisphere, due to well-known lateralization of speech mechanisms (Albouy et al. 2020; Fedorenko et al. 2024; Hickok and Poeppel 2007), we also conducted ROIs analysis within the right hemisphere to control for the hemispheric specificity of our effects. For univariate analyses (Table 4), we found no effects of social context in any of the right ROIs, and only trends for an effect of semantic context (higher activity for scrambled than intact conversations) in R_IFG, R_MFG and R_aSTS. We also found a trend for an interaction between these two factors (higher difference of activity between intact and scrambled for dialogues than monologues) in R_aSTS only. Overall, we didn’t find any significant univariate effects in the right hemisphere, with only some trends mimicking the effects found in the left hemisphere.

**Table 4:** Results of the 2 social (dialogues/monologues) X 2 semantic (intact/scrambled) ANOVAs within functionally localized right ROIs.

ROIs	Effects of social			Effects of semantic			Interactions		
	*F*(1,23)	*P*	*np2*	*F*(1,23)	*P*	*np2*	*F*(1,23)	*P*	*np2*
R_IFG	0.15	> 0.250	0.01	3.48	0.076	0.21	2.71	0.114	0.11
R_MFG	< 0.01	> 0.250	< 0.01	3.81	0.063	0.19	0.81	> 0.250	0.03
R_aSTS	1.64	0.214	0.15	3.70	0.067	0.10	3.57	0.072	0.14
R_pSTS	0.85	> 0.250	0.04	2.84	0.105	0.10	2.82	0.107	0.11
R_AG	0.83	> 0.250	0.05	0.31	> 0.250	0.02	< 0.01	> 0.250	< 0.01
R_A1	0.08	> 0.250	< 0.01	1.55	0.226	0.05	0.13	> 0.250	0.01

Furthermore, when using MVPA to test single-sentence representations as a function of the context in the right hemisphere, we found a main effect of social context in the R_AG, with no other significant effects or interactions (Table 5).

**Table 5:** Results of the 2 social (dialogues/monologues) X 2 semantic (intact/scrambled) ANOVAs for the MVPA decoding accuracies within functionally localized right ROIs.

ROIs	Effects of social			Effects of semantic			Interactions		
	*F*(1,23)	*P*	*np2*	*F*(1,23)	*P*	*np2*	*F*(1,23)	*P*	*np2*
R_IFG	0.07	< 0.250	<0.01	0.80	> 0.250	0.03	0.46	< 0.250	0.02
R_MFG	0.02	< 0.250	<0.01	1.11	> 0.250	0.03	3.01	0.096	0.12
R_aSTS	2.67	0.116	0.08	0.19	> 0.250	0.01	0.27	< 0.250	0.01
R_pSTS	< 0.01	> 0.250	<0.01	0.24	> 0.250	0.01	0.24	< 0.250	0.01
R_AG	6.18	**0.021**	0.11	0.91	> 0.250	0.02	0.14	< 0.250	0.01
R_A1	0.51	> 0.250	0.02	0.24	> 0.250	0.01	0.50	< 0.250	0.02

## Discussion

We investigated how semantic and social contexts shape neural mechanisms of speech processing, using 7 T fMRI to measure responses to dialogues and monologues that were either semantically intact or sentence-scrambled. Importantly, all individual sentences were semantically coherent, meaning the conversation-level dynamics modulated the semantic or social relationships only across sentences. Building on our previous behavioral study (Abassi and Zatorre 2025), which showed that listeners process sentences more efficiently in dialogues than monologues and in intact than scrambled conversations, we examined the corresponding neural mechanisms. Left pSTS showed stronger univariate activity for scrambled versus intact conversations, consistent with predictive coding accounts of language. This effect was enhanced for dialogues relative to monologues, reflecting increased violation of expected turn-taking when conversational coherence was disrupted. In parallel, left aSTS and IFG exhibited higher multivariate decoding accuracy for sentences in dialogues than monologues, indicating sharper neural representations. Together, these results show that social interaction enhances neural representations, whereas semantic disruption amplifies prediction-error signals, an important dissociation that clarifies how social and semantic contexts jointly modulate auditory speech processing.

Our univariate findings align with prior work linking the STS to semantic processing (Hickok and Poeppel 2007; Friederici 2012; Fedorenko et al. 2024) and to predictive mechanisms during speech processing (Sohoglu et al. 2012; Broderick et al. 2019). Here, increased activity for sentence-scrambled over intact conversations in left pSTS (Figs. 2 and 3) suggests sensitivity to violated semantic expectations, consistent with prediction error mechanisms (Rao and Ballard 1999; Friston 2005; Clark 2013). This outcome supports the idea that the brain continuously generates top-down predictions about upcoming linguistic input, a core mechanism during speech processing (Shain et al. 2020; Heilbron et al. 2022; Caucheteux et al. 2023). Similar effects have been observed by Landsiedel and Koldewyn (2023) for scrambled over intact dialogue. Whereas traditional models posit greater activation for structured input due to coherence (Friederici 2012; Hagoort 2017), predictive coding offers an alternative perspective: structured input reduces error while disruption increases demand on predictive systems. Our findings extend these principles beyond sentence-level to multi-sentence conversational contexts, indicating that the STS not only predicts upcoming linguistic content within a sentence, but also maintains expectations for cross-sentence conversational structure. This effect supports the proposed role of the STS in integrating auditory and linguistic cues over time (Hasson et al. 2007; Sohoglu et al. 2012). Notably, this context-driven modulation occurred for identical target sentences, confirming that the differences in neural activity arose solely from context rather than content. Thus, STS contributes to maintaining and updating mental representations of ongoing conversations, a key function for natural speech comprehension.

Although there was no main effect of social context on univariate activity, an interaction emerged between social and semantic factors, with scrambled dialogues eliciting the strongest responses (Figs. 2 and 3). This pattern involved a network including STS, IFG, and MFG, region implicated in both speech perception and higher-order language processing (Fedorenko et al. 2024). These findings suggest that social context modulates semantic structure processing, especially when that structure is violated. One explanation may be that, in dialogues, listeners expect turn-taking, mutual coherence, and shared semantic context. When this structure is violated through sentence scrambling, the mismatch between expectation and input become more salient, eliciting increased neural effort to resolve ambiguity. In monologues, by contrast, each sentence may be treated as relatively self-contained, reducing reliance on inter-sentence predictions and reducing the impact of scrambling. Thus, dialogues may amplify prediction-related processing, making semantic violations more disruptive and neural representations more salient. This interpretation is consistent with evidence that social interaction enhances perceptual sensitivity to social cues (Dikker et al. 2014; Redcay and Schilbach 2019), and that conversational dynamics engage predictive mechanisms distinct from those used in isolated speech (Stolk et al. 2013; Pickering and Garrod 2021), allowing listeners to anticipate upcoming speech patterns based on conversational context. Related to this point, we note that our monologues were following the exact same structure that our dialogues, raising the question of whether they were closer to resembling a single-person dialogue than following pragmatic conventions of what usually constitutes a monologue (as seen in a sermon, lecture or oral arguments in court). However, we highlight that our goal was not to construct canonical monologues, but to create non-interactive versions of the very same dialogues while keeping all linguistic content strictly matched across social conditions. Thus, while this aspect may make some monologues sound less conventional, it did not affect the intended contrast between interactive and non-interactive speech.

Our MVPA results further highlight the influence of social cues on speech encoding, showing better sentence classification in dialogues than monologues, particularly in aSTS and IFG. Importantly, this advantage occurred regardless of whether the dialogue was intact or scrambled, indicating a robust facilitation by social context alone. These findings mirror our previous behavioral results (Abassi and Zatorre 2025) and are also coherent with theories of interactive alignment in dialogues (Pickering and Garrod 2004, 2021; Branigan et al. 2011; Menenti et al. 2012). Notably, while univariate analysis showed higher activation for scrambled as compared to intact dialogues, MVPA revealed better decoding for both intact and scrambled dialogues compared to monologues. This dissociation, stronger BOLD response without improved neural representational, provides novel support for the predictive coding framework. In this view, prediction errors (eg from violated semantic context) would increase neural response magnitude without necessarily improving neural representation. Indeed, while prediction error signals are generally strongest when expectations are violated, they do not necessarily encode the content of the violation itself. Instead, they reflect the presence of a mismatch, with representational content carried separately by distributed neural patterns (Caucheteux et al. 2023; Heilbron et al. 2022; De Lange et al. 2018). Conversely, the dialogue advantage likely reflects a different kind of prediction, driven by social interaction, arising from turn-taking and the expectation of alternating agents rather than from semantic coherence. Such socially driven sharpening aligns with frameworks of the social brain, emphasizing predictive coding of others’ communicative actions and expressions (Tamir and Thornton 2018; Lu et al. 2025).

This division of roles suggests a functional dissociation between regions and mechanisms: posterior STS activity increases with disrupted predictions, while decoding improvements in dialogues in anterior STS and IFG reflect greater contextual facilitation of sentence representation. This distinction underscores the multi-layered nature of predictive processing, balancing error signaling and encoding fidelity depending on context. Overall, the aSTS and IFG emerged as key hubs in our multivariate results. The aSTS has previously been implicated in hierarchical integration of linguistic structures over time (Bornkessel-Schlesewsky and Schlesewsky 2013), making it well-suited to encode discourse-level features such as conversational flow. The IFG, in turn, is known for predictive language processing in various contexts (Friederici et al. 2006; Rodd et al. 2010, 2013; Friederici 2011). Our findings suggest that, while both the posterior and anterior STS activity tracks contextual violations, its anterior portion as well as the IFG support the sharpening of sentence representation within a social context. This division of labor may underline the brain’s ability to flexibly adapt to conversational structure, engaging predictive mechanisms when coherence is present and elevating error signaling when it is violated.

The observed effects were predominantly left-lateralized, consistent with established left-hemisphere dominance for speech and language (Albouy et al. 2020; Fedorenko et al. 2024; Hickok and Poeppel 2007). Right hemisphere homologues showed weaker trends, suggesting that fine-grained linguistic predictions are primarily left-lateralized, whereas prosodic or global structural cues may rely more on the right hemisphere (Sammler et al. 2015). Future work could examine this lateralization more directly by manipulating prosodic cues within conversations. On another aspect, while our stimuli were controlled for prosody and voice characteristics, real-world conversations involve additional layers of complexity, including non-auditory cues (eg gestures, facial expressions). Incorporating such multimodal features could provide a more comprehensive understanding of how social and semantic contexts interact in natural settings (Holle et al. 2010; Puschmann et al. 2019; Olson et al. 2023).

## Conclusion

Overall, our study reveals the intricate interplay between semantic and social contexts in shaping neural responses to conversational speech. By demonstrating that dialogues enhance sentence processing in speech-selective brain regions, we provide evidence for specialized neural mechanisms that support speech comprehension in quasi-naturalistic settings. The improved classification accuracy in dialogues vs. monologues observed in our study supports the idea that the brain actively leverages social and contextual cues to optimize speech processing. This may reflect a domain-general representational sharpening under social contexts. Indeed, converging evidence from vision research shows that socially relevant stimuli enhance perceptual discrimination and neural encoding. For instance, the presence of interacting agents improves sensitivity to social objects and their relations (Papeo et al. 2019; Goupil et al. 2024) and leads to sharper cortical representations in the visual cortex (Abassi et al. 2020; Bellot et al. 2021). These parallels suggest that social interactions may universally enhance the efficiency of perceptual systems, perhaps by heightening attention, boosting prediction, or increasing the salience of behaviorally relevant stimuli. Extending this perspective, our findings raise the possibility that predictive and sharpening mechanisms observed in dialogue processing reflect domain-general neural strategies for optimizing information processing in social contexts. Future research could build on these findings to identify how individual differences, such as social communication skills, could influence the integration of semantic and social information. Together, these efforts will contribute to a more comprehensive understanding of the cognitive and neural foundations of verbal human communication.

## Supplementary Material

stims_sample_bhag023

## Data Availability

Audio stimuli, second level fMRI maps and preprocessed fMRI data are available online at https://osf.io/vqs7h/.
